# Learning How Preoperative Communication Relates to Postoperative Experiences for Older Veterans Having Inguinal Hernia Surgery

**DOI:** 10.1111/jgs.70216

**Published:** 2025-11-26

**Authors:** Melissa A. Thornton, Elisa L. Marten, Nicole Lunardi, Don Mai, Cameron Macdonald, Jocelyn G. Baker, Ava Hitzeman, Celette Sugg Skinner, Cynthia J. Brown, Miles Berger, Simon Craddock Lee, C. Munro Cullum, Konstantinos I. Makris, Thai H. Pham, Aanand Naik, Victoria Tang, Courtney J. Balentine

**Affiliations:** 1 Department of Surgery, University of Texas Southwestern Medical Center, Dallas, Texas, USA; 2 VA North Texas Health Care System, Dallas, Texas, USA; 3 Wisconsin Surgical Outcomes Research, Madison, Wisconsin, USA; 4 William S. Middleton Memorial VA Hospital, Madison, Wisconsin, USA; 5 Department of Surgery, University of Wisconsin-Madison SMPH, Madison, Wisconsin, USA; 6 Qualitative Health Research Consultants, LLC, Madison, Wisconsin, USA; 7 Peter O’Donnell Jr. School of Public Health, University of Texas Southwestern Medical Center, Dallas, Texas, USA; 8 Department of Internal Medicine, Louisiana State University School of Medicine, New Orleans, Louisiana, USA; 9 Department of Anesthesiology, Stanford University, Palo Alto, California, USA; 10 Department of Population Health, University of Kansas Medical Center, Kansas City, Kansas, USA; 11 Department of Psychiatry, University of Texas Southwestern Medical Center, Dallas, Texas, USA; 12 Department of Surgery, Baylor College of Medicine, Houston, Texas, USA; 13 Michael E. DeBakey Veterans Medical Center, Houston, Texas, USA; 14 Division of Geriatric Medicine, University of Texas Health Science Center, Houston, Texas, USA; 15 Department of Internal Medicine, University of Texas Health Science Center, Houston, Texas, USA

**Keywords:** hernia surgery, postoperative recovery, qualitative research, surgery experience, veteran

## Abstract

**Background:**

For older adults to decide whether inguinal hernia repair will meaningfully improve their lives, it is critical to (1) understand how the operation affects them and whether it enhances outcomes that matter to them, and (2) identify ways to improve how surgeons discuss the benefits of surgery and how they prepare older adults for postoperative recovery.

**Methods:**

We conducted semi-structured interviews with 40 Veterans ≥ 65 years old who had inguinal hernia repair at two high-volume Veterans Affairs hospitals.

**Results:**

Participants were all men; their mean age was 73 years, 65% were White, and 33% were Black. Older adults felt that the surgical team provided excellent reassurance regarding the safety and efficacy of surgery but expressed a desire for improved listening during preoperative counseling and for clearer communication regarding the reality of postoperative recovery. Veterans reported a return to baseline physical and cognitive function between 2 days and 6 weeks after surgery, though two Veterans experienced significant short-term cognitive dysfunction. Those who reported dissatisfaction with preoperative communication were more likely to be surprised or concerned about postoperative symptoms.

**Conclusions:**

Our study provides critical information on how hernia repair affects the lives of older adults, and this can be used to better prepare the patients for surgery and to help them decide whether surgery will meaningfully enhance their quality of life.

## 1 | Introduction

Each year in the United States, approximately 250,000 adults aged 65 years and older undergo surgical repair of an inguinal hernia [1]. Although inguinal hernia repair is the most common elective general surgery procedure for older adults, most studies in the surgical literature have focused on how surgery affects outcomes such as morbidity, mortality, and hernia recurrence [2–9]. Although these outcomes are certainly important, it is equally valuable to have an in-depth understanding of the ways in which hernia surgery affects the day-to-day lives of older adults. Surgery, even in the absence of discrete complications, can induce changes that affect functional independence, quality of life, and the ability to live at home. Additionally, it is critical to understand what older adults hope and expect to gain from surgery that balances any risks of the operation. When surgeons do not understand or have misconceptions about how older patients experience preparation for surgery and postoperative recovery, several significant problems can arise. First, patients may experience considerable regret and dissatisfaction over their treatment decision if the outcomes they expect from surgery differ significantly from what they actually experience [10–12]. Second, many postoperative issues and complications can be effectively managed or prevented when patients and care teams are adequately prepared to address them, but lead to readmissions, emergency room visits, and multiple calls to the care team when they are unexpected [10–12]. Third, from an ethical standpoint, informed consent depends on the provision of accurate information to patients enabling them to make reasoned decisions about whether to have an operation and which operation they desire. When surgeons are unable to accurately portray the profile of risks and benefits from an intervention, including how surgery can affect patients’ lives beyond the impact of discrete complications, it becomes impossible to meet our ethical obligation to patients. Having high-quality, and in-depth qualitative information on this aspect of surgical recovery can therefore help improve the quality of conversations about whether and when to proceed with surgery.

The purpose of this study was to (1) explore the lived experience of patients ≥ 65 years of age who have undergone inguinal hernia repair, and (2) identify ways to improve preoperative communication about how surgery affects the lives of older adults. Identifying gaps in communication and providing critical information to help patients understand and prepare for hernia surgery are both necessary to increase the quality of postoperative care for older adults.

## 2 | Materials and Methods

### 2.1 | Design

This is a multi-site descriptive qualitative research study that used semi-structured virtual interviews to explore pre-, peri-, and postoperative experiences following inguinal hernia repair for patients 65 years of age and older. Interviews lasted from 60 to 90 min in duration.

### 2.2 | Setting

The study was conducted at two high-volume VA hospitals: (1) VA North Texas Health Care System (VNTHCS) in Dallas, TX, and (2) the Michael E. DeBakey VA Medical Center (MEDVMC) in Houston, TX.

### 2.3 | Ethical Considerations

Prior to conducting the study, we were granted Institutional Review Board (IRB) approval from the University of Texas Southwestern Medical Center, Baylor University, and the University of Wisconsin-Madison. Additionally, the study obtained endorsement from the VA Research and Development Committee (R&DC) of the VNTHCS, MEDVMC, and William Middleton VA. All components of the study’s design and completion adhered to the principles set forth in the.

EQUATOR network’s Standards for Reporting Qualitative Research [13, 14].

### 2.4 | Participants

Using purposive sampling, participants were recruited from the VNTHHCS and MEDVMC between January 2022 and June 2023. Patients were eligible for inclusion if their age was ≥ 65 years and they completed surgical repair of a unilateral, non-recurrent inguinal hernia within the prior 2 months. Patients were excluded if they had cognitive deficits that would interfere with interview participation or did not speak English. The planned sample size for the study was 40 participants, stratified by hospital, age, and type of anesthesia used during surgery.

### 2.5 | Interview Guide

An interview guide was developed for the study (Table S1), which included open-ended questions and prompts about the inguinal hernia repair experience and postoperative recovery, as well as an exercise in rating how concerned Veterans would be given hypothetical postoperative scenarios. Questions for the interview guide were designed to explore outcomes that are important to older patients (such as cognitive and physical recovery, ability to maintain independence, etc.) and were based on surgeon experience, review of the literature, and preliminary discussion with older Veterans. Pilot interviews were conducted with four Veterans and the interview guide was revised based on their feedback. Interview guides also explored additional topics that are not reported in this paper.

### 2.6 | Data Collection

Approved research personnel screened charts for eligible Veterans who were then approached in surgery clinics during their initial preoperative surgical consultations. Veterans who elected to participate signed an informed consent and HIPAA authorization form at that time. After consent, participants were referred to an independent research firm, Qualitative Heath Research Consultants (QHRC), to schedule interviews between 2 and 8 weeks after their inguinal hernia repair. Trained qualitative interview specialists contacted the Veterans on their scheduled interview date and conducted telephone interviews that focused on their perioperative experiences and postoperative recovery following their recent inguinal hernia repair. Interviewers started each interview by reading an informational sheet and completing verbal consent. The Veterans were also reminded of the regulatory guidelines in place to protect their personal health information and privacy. Upon completion of the interview, the Veterans were compensated $50. Interviews were audio-recorded, transcribed verbatim, and de-identified by QHRC following HIPAA guidelines to ensure Veteran privacy and confidentiality.

### 2.7 | Data Analysis

Research assistants, the QHRC Principal, and the study principal investigator used Conventional Content Analysis to open code 20% of all interview transcripts to produce a thematic codebook [15]. The generated codebook themes were then applied to all transcripts using NVivo qualitative analysis software [16]. All transcripts were coded by three research assistants, with every fourth transcript being commonly coded to verify intercoder consistency. This resulted in a *kappa* score of 0.81, which demonstrated excellent agreement. The research team held frequent meetings to discuss emerging themes in the interviews and to adjudicate any coding disagreements. The most frequently occurring themes regarding satisfaction and dissatisfaction with the surgical journey are discussed below.

## 3 | Results

### 3.1 | Participant Demographics

All participants were men, the mean age was 73 ± 5.2 years, 65% were White, and 33% were Black (Table 1). Hernias were repaired using an open Lichtenstein approach in 38 (95%) Veterans, and two (5%) hernias were repaired laparoscopically. Surgery was performed using local anesthesia for 19 (48%) Veterans, while 18 (45%) received general anesthesia. Additionally, three Veterans began their hernia repairs under local anesthesia, but the surgeon converted to general anesthesia.

### 3.2 | Interview Themes

The semi-structured interviews identified several common themes that arose during the pre- and postoperative periods of inguinal hernia repair for older adults. These included positive and impactful experiences but also identified facets of the surgical journey that could be improved.

### 3.3 | Preoperative Experiences

#### 3.3.1 | Appreciating Reassurance From the Surgical Team

As even a simple surgery can be stressful for older patients and their families, many participants expressed appreciation for the substantial reassurance they received from their surgical team regarding the safety and efficacy of hernia surgery. Veterans described feeling comforted and confident in the care provided, which helped alleviate preoperative anxiety and built trust. To exemplify this theme, one Veteran shared:

That’s another thing I was impressed with, is … three people on the team came out, the head nurse and then the anesthesiologist and then … the surgeon. And they each talked to me about what their little game plan was. And I think that’s really comforting to a patient, that he knows that all of them sit there and go through what they’re fixing to do. (Veteran 1)

His insights underscore the value of dedicating time to adequately inform, counsel, and reassure patients about the planned treatment.

#### 3.3.2 | Frustrations With Communication

Although many older Veterans expressed that their surgeons did an excellent job of providing general reassurance regarding surgery, several also stated that communication could be improved by providing more specific details about what to expect on the day of their operation and during the subsequent postoperative recovery. Participants reported feeling uncertain and uninformed about many aspects of their surgery, including how to best prepare for the operation, whether there were alternative surgical and anesthesia approaches in addition to the ones they were offered, and what they could expect for postoperative care and time to full recovery. Veterans repeatedly emphasized the need for clearer and more comprehensive communication from their surgical team. As one frustrated Veteran described,

Either the staff is ignorant of what you should do, or they’re complicit. They didn’t tell me how to conduct myself after surgery. I just had to figure it out myself. (Veteran 2)

This illustrates how Veterans can feel inadequately informed and prepared for the postoperative recovery process even when they feel a general sense of reassurance about the operation and are comfortable with their surgeons.

#### 3.3.3 | The Importance of Listening

In addition to the need for better preoperative communication about more specific details of postoperative care and recovery, Veterans also highlighted the importance of healthcare providers actively listening to their concerns, preferences, and prior surgical experiences when discussing the risks, benefits, and alternatives to hernia surgery. They expressed a desire for greater empathy and understanding from the surgical team, particularly in addressing their emotional and physical needs related to the hernia. Veterans stated that a more patient-centered approach would enhance their overall satisfaction with the surgical experience. When one Veteran was asked if he was able to express his concerns with the surgical team preoperatively, he stated:

Not that in depth. I mean, I told them I was [a] little apprehensive about it and scared…And they - you know - ‘You’ll be okay, you’ll be okay, everything is going to be fine, done this a million times, don’t worry about it. Blah, blah, blah…’ (Veteran 3)

Additionally, some Veterans recalled being given the option between receiving local or general anesthesia, but others did not. For example:

… the dude that [came] in and told me, he’s going to use a local. That was the first time I was a little bit concerned because I wanted to be out. But he told me he going to use local and I’d feel better, and I’d thank him for it when I get out of surgery because I wouldn’t have a headache later on in the day. (Veteran 4)

On the other hand, one Veteran described having a notably positive experience with his care team due to their attentiveness of to his needs and experience:

That place and those people there are super kind and friendly. It’s very, very clean, well-organized, and they meet their appointments most of the time, always on time. And, I mean, they ask questions, and they intently listen, and they make their notes and do their assessments. And you leave that place, your primary care … person there, she knows a little bit about you. (Veteran 5)

Veterans also wanted their surgical team to better understand how the hernia surgery might affect their quality of life, particularly regarding the psychosocial impacts of surgical recovery, and expressed a desire to be seen and heard beyond their medical diagnosis. Respondents indicated that when discussing the risks and benefits of surgery, the surgeons tended to focus primarily on the technical aspects of the planned operation and potential complications but spent considerably less time eliciting and discussing Veterans’ concerns about how the operation could affect other aspects of their lives. Additional illustrative quotations regarding the preoperative experience are shown in Table 2.

### 3.4 | Postoperative Experiences

#### 3.4.1 | Postoperative Pain and Discomfort

Despite many preoperative concerns and questions about postoperative pain, few Veterans recalled notable pain or discomfort beyond the first few days of their recovery. Several did note being surprised by the degree of significant pain during the first few days after surgery. However, when asked more specifically about whether pain or discomfort limited their activities, most Veterans described their pain as well controlled with medication, and stated that it did not notably affect their ability to perform daily activities or exercise after the immediate recovery period.

In particular, Veterans who expressed satisfaction regarding pre-operative communication with their care team often felt subjectively better prepared to manage their post-surgical pain levels. As one Veteran explained:

They told me that there was going to be possible pain after the surgery, but for me, I felt it was just more soreness than pain. So, I’ve been doing alright with it. (Veteran 6)

Conversely, Veterans who initially reported instances of poor communication with their care team prior to surgery often stated that they felt subjectively unprepared for their experience of post-surgical recovery. As one Veteran recalled:

Well, they didn’t exactly say there would be much pain involved. They said it’s pretty standard. You’ll be sore for a couple days, and then you’ll be okay. And that’s not exactly true (laughs). And …. I don’t deal [well] with people lying to me and trying to throw wool over my eyes. (Veteran 7)

This quote highlights how some Veterans may feel deceived or misled by their surgeons if care is not taken to address the full range of potential experiences of postoperative pain and discomfort so that patients better understand what to expect after surgery.

#### 3.4.2 | Cognitive Dysfunction After Surgery

Since exposure to surgery and anesthesia can potentially lead to short- and long-term cognitive changes in older patients, we explored whether our Veteran participants had noticed any significant cognitive dysfunction following their operations. Most Veterans reported minimal, if any, notable cognitive changes following surgery, aside from the immediate postoperative period. Veterans who did note postoperative challenges with memory, concentration, or other cognitive functions generally described this change as being of short duration (i.e., hours rather than days). One Veteran described his postoperative cognitive changes as:

…when I was looking at [discharge instructions] and trying to read it, it wasn’t making all that much sense…that was, that was pretty much until I got home. Maybe I had been home maybe a couple of hours before my mind actually cleared up. (Veteran 8)

However, patients with preoperative cognitive concerns reported longer periods in which they experienced worsened postoperative cognitive change. This is exemplified by an exchange between one Veteran and his wife during an interview:

Veteran:

Come answer … a question… Hey, that confusion, you know, head confusion, I mean, it hasn’t get worse after I had the surgery? I had it before I had the surgery.

Veteran’s wife: After…

I Veteran: It wasn’t before?

I Veteran’s wife: Not as bad as it is now. (Veteran 9)

One proposccidence of postoperative cognitive dysfunction in older patients is to use alternatives to general anesthesia, such as performing surgery under local anesthesia.

Since our sample was designed to be equally divided among Veterans who had surgery with local and general anesthesia, we probed whether there was any subjective difference in perceived postoperative cognitive function between these two groups. Our interviews did not reveal any differences in the duration or types of postoperative cognitive changes following surgery with local or general anesthesia. In fact, most patients in the local anesthesia group were unaware that they had not received general anesthesia, as they remembered very little about their actual operation.

#### 3.4.3 | Postoperative Return to Normal

One of the common questions that arises during preoperative counseling with older patients is how long it will take to recover or return to baseline after surgery. When older Veterans were asked to describe when they felt that they had returned to their normal preoperative level of function, most of our participants felt this occurred by 6–8 weeks after surgery. As one Veteran explained:

[I’m] almost normal, but I still don’t do no heavy lifting or too much straining. I’m going to wait about six weeks. This is about five weeks now, something like that. (Veteran 9)

We found in discussion that many Veterans defined their “return to normal” as the ability to resume regular exercise or participate in physical activity around their homes such as lawn work.

#### 3.4.4 | The Primary Barrier to Recovery—Lifting Restrictions

Despite being generally satisfied with the trajectory of normal recovery, Veterans frequently expressed frustration with the postoperative lifting restrictions imposed on them by their surgeons. These restrictions were viewed as their main barrier to full physical recovery, and multiple Veterans questioned whether they were truly necessary. Despite these frustrations, Veterans generally described following lifting restrictions for the duration requested by their surgeons and found other ways to stay active that did not violate this recommendation. Demonstrating this common interview theme, one Veteran described his postoperative recovery as:

…100 percent. Except for lifting, you know, I’m still a week away from being able to lift 10 pounds. But I found out that I could play tennis and I could exercise, so I’m playing tennis and exercising. (Veteran 10)

Of note, 21/40 Veterans described themselves as “more active” than other people their age, and their higher levels of activity may have contributed to their frustration when surgeons instructed them to limit physical activity postoperatively.

#### 3.4.5 | The Effect of Comorbidities on Recovery

An additional theme that arose organically from the interviews was how comorbidities affected the experiences of older Veterans, especially after surgery. Although Veterans felt that their preoperative health conditions greatly affected the trajectory of physical recovery, they also expressed that any difficulties stemming from these comorbidities were expected, and they did not blame the surgeons or the surgery for this. One Veteran with a history of chronic obstructive pulmonary disease (COPD) explained how this comorbidity affected postoperative recovery by stating,

I go fishing, and I wasn’t doing a lot. I couldn’t get out and mow the grass. You know, I’ve got COPD, so I get short of breath real[ly] easy. So, I don’t get out and work in the yard… (Veteran 11)

Another Veteran discussed how his history of cerebrovascular accident affected postoperative recovery when asked how back to normal he felt on a scale of 1 to 10 (with “10” being his baseline function). He stated,

I say about an 8, because I still feel like I’m not a 10, because, maybe because of the stroke, but I feel like I’m about an 8. (Veteran 12)

Additional illustrative quotations regarding postoperative experiences are shown in Table 3.

## 4 | Discussion

In this qualitative evaluation of the pre- and postoperative experiences of older Veterans undergoing inguinal hernia repair, we identified multiple ways to enhance preparation for and recovery from surgery for older adults by improving preoperative communication through a better understanding of how surgery impacts patients’ lives. Importantly, we also found that patients who reported greater satisfaction with the quality of preoperative communication also subjectively felt more prepared to manage postoperative recovery and felt better about their postoperative pain.

We found that surgeons excelled at reassuring older Veterans about the overall safety and effectiveness of inguinal hernia surgery during the initial consultation. However, preparation for surgery could be enhanced by providing more specific and actionable information about how surgery and anesthesia might affect patients’ lives. In particular, we found that patients strongly desired more information on what they could do to prepare for surgery, and on how surgery and anesthesia can affect physical function, cognitive function, and their normal daily activities. Our interviews provide a template for approaching this discussion by exploring the variety of ways that inguinal hernia repair can affect these important outcomes and by highlighting the extent of inter-patient variability and how pre-existing comorbidity can change the postoperative experience. Although surgeons typically spend a great deal of time explaining the technical aspects of the operation and the risk of the most common and significant complications, our work is consistent with several prior studies showing that surgeons are less adept at explaining how the operation affects key aspects of patients’ lives [17–22]. Additionally, older Veterans placed great weight on communication and building rapport with their surgical team. Our interviews showed that spending more time listening to patient concerns and expressing empathy represents another substantial opportunity for improvement.

Several studies have shown that setting expectations about outcomes such as postoperative pain may be effective at reducing the need for opioid medications after surgery and can actually lead to reduced levels of perceived pain [10–12, 23, 24]. Comments from our participants lend some support to this concept.

Additionally, older adults frequently express concern about how surgery and anesthesia affect cognitive function, and surgeons should be prepared to discuss how hernia repair can influence cognition and discuss strategies for mitigation. We found that significant cognitive dysfunction for most older Veterans was of relatively short duration in the absence of major comorbidities, but older patients with a higher burden of comorbidity may expect to experience more frequent and longer bouts of cognitive dysfunction. For these patients, it may be relatively more important to involve their families and caregivers in postoperative care to avoid any difficulties that might arise from more prolonged or pronounced cognitive dysfunction.

Regarding physical recovery, we found that the trajectory of return to normal function after inguinal hernia repair was typically complete by around 2 months in older Veterans. In fact, the primary speed bump on the road to recovery seems to be externally imposed when surgeons ask their patients to avoid lifting heavy objects for several weeks after surgery. A recent review of practice patterns for high-volume hernia surgeons showed that despite the lack of strong evidence supporting lifting restrictions after hernia repair, the vast majority of surgeons instruct patients to avoid lifting “heavy” objects for anywhere from 2 to 6 weeks after surgery [25]. Given the concerns expressed by our interviewees and the lack of evidence supporting any benefit to postoperative weight restrictions, it is worthwhile to ask whether this practice could reasonably be abandoned or at least limited to a shorter period of time.

### 4.1 | Limitations

Although our interviews with older adults identified several concrete steps that could improve perioperative care, there are several limitations of our study. First, our population of older, male, and English-speaking Veterans may have very different peri- and postoperative experiences than younger patients, non-Veterans, women, and individuals with limited English proficiency. However, the vast majority of patients with inguinal hernias are men, and the themes that emerged from our interviews seem, at face value, highly applicable to the general population of individuals with inguinal hernias regardless of biological sex or military background. We also only interviewed participants after surgery, which could introduce recall bias and did not conduct a quantitative analysis of quality of life for comparison. Additionally, Veterans may have been reluctant to discuss negative opinions or experiences with our research team, making our assessment of their recovery overly optimistic. To address this concern, all interviews were conducted by an independent qualitative research firm that has no affiliation with the VA or the surgical teams who operated on participants. The value of having independent interviewers was highlighted by their ability to elicit criticisms about preoperative communication and postoperative restrictions on physical activity. Further, although we noted that there were some subjective differences in how patients who were more or less satisfied with preoperative communication tended to describe their postoperative experiences, this was not a statistical comparison and should be regarded as an exploratory evaluation in the context of literature linking quality of communication with postoperative outcomes. Similarly, other comparisons were based on purely qualitative data, so it was not possible to quantify and conduct statistical tests. However, in the context of understanding the perspectives of older adults, using qualitative data is not only reasonable, but can provide considerably more depth and insight into the patient experience (and ways to improve this experience) than administering a simple survey that yields a series of numbers without any understanding of what drives the score on a given instrument. Qualitative interviews give both context and detail into why certain aspects of care were positive, and why other parts were negative.

## 5 | Conclusions

Our findings provide a road map to potentially enhance peri- and postoperative recovery for older adults having inguinal hernia surgery by improving the quality of preoperative communication to incorporate a richer and more detailed conversation about the practical impact of hernia repair on the lives of older adults.

## Supplementary Material

supplemental

Additional supporting information can be found online in the Supporting Information section. Data S1: Supporting Information.

## Figures and Tables

**TABLE 1 |:** Veteran participant demographics.

	*N* (%)
Age in years	73 (5.2)
Male sex	40 (100%)
Race	
White	26 (65%)
Black	13 (33%)
Unknown	1 (2%)
Surgical approach	
Open	38 (95%)
Laparoscopic	2 (5%)
Anesthesia approach	
Local	19 (48%)
General	18 (45%)
Local converted to general	3 (7%)

*Note:* Values represent number (%) for categorical variables and mean (standard deviation) for continuous variables.

**TABLE 2 |:** Quotes of Veteran perspectives on the perioperative experience.

Theme	Veteran quote 1	Veteran quote 2
Appreciating reassurance from the surgical team	*I talked with him, and I felt, you know, reassured that he’s going to do a good job. (Veteran 13)*	*I was quite pleased with the way they came in, kept me informed, and, you know, everybody that was any participation in the surgery came in and explained exactly what they were going to do and how it was going to work. (Veteran 14)*
Frustrations with communication	*Something as simple as, “you might have bruising,” would’ve been all I needed to hear*… *would’ve been nice if somebody had told me*. *(Veteran 15)*	*They hand me the paper with all the details of the dos and don’ts, and it’s up to you to read it … They probably could spend, you know, 3–5 min with each-, going over that with you to make sure that you fully understand that rather than just handing you the paper and say, here’s the dos and don’ts*. *(Veteran 16)*
Importance of listening	*Sometimes doctors really didn’t seem to listen to exactly what you were saying*. *(Veteran 17)*	*They didn’t understand what I was saying. (Veteran 13)*

**TABLE 3 |:** Quotes of Veteran perspectives on the postoperative experience.

Theme	Veteran quote 1	Veteran quote 2
Efficient care on the day of surgery	*I went out. I went under. And I popped up just like they said, you know, 2 h later. And I was feeling pretty good, no complications on the anesthetic at all. So, it worked out really well. (Veteran 1)*	*I woke up and, you know, it just went*… *came out good. I guess, same thing, you know, I remember moving over on the bed, and then, boom!* …*I woke up in the place I started. I didn’t have any problems. (Veteran 18)*
Postoperative pain or discomfort	*After I came out under the … anesthesia and everything, the pain, it really wasn’t as excruciating as I thought it—well, I was sore and everything, but*…*I was kind of expecting something a little worse. (Veteran 19)*	*I didn’t know what to expect. I mean… I didn’t know it was going to be so painful afterwards. (Veteran 20)*
Cognitive dysfunction after surgery	*No [confusion]. No, they came in. They said, “How do you feel?” I feel fine. I says, “Can I get dressed?” They said, “Yep, go ahead, get dressed, and you can leave.” I got dressed, and I left. (Veteran 21)*	*Not even [confused] on the ride home or not even after I got woke up out of surgery. I was a little foggy when I first woke up. Got that first cup of coffee in me, and I was all right. I was fine. (Veteran 22)*
Postoperative return to normal	*Well, uh, after the 3 days, you know, uh, I start to get up and down from the bed easier. And then, then after the 1 week, and you know, each week. Now the third week, I can, you know, pretty much pop up and down, no problem at all*. *(Veteran 18)*	*It’s just been this week that I’ve kind of felt like I’m back at 100%, and it has been, I think, 7 weeks now. (Veteran 23)*
The main barrier to recovery—lifting restrictions	*Well, not at all [back to normal], because I can’t do the same exercise routine…every other day, I would lift weights for an hour…even with what the doctor told me I could do, that’s going to be boring and depressing, and I’m not going to be able to do some of the things that I wish I could do, because I’m not going to do anything stupid.…I know I won’t be to that point probably for at least another month*. *(Veteran 24)*	*I think it was fine. They did a good job. I’m happy. I’m better. You know, another two, 3 months, I’ll be back to lifting whatever I need to lift without having to worry about it popping, or hopefully. (Veteran 17)*
The effect of comorbidities on recovery	*[Back to normal] like about 80%. It’s just the gout. The gout is going to be there. Like today I’m having a bad day with my gout and it’s swelling of the feet, you know*. *(Veteran 25)*	*Nah, [activity level] is about the same*…*I don’t do a whole lot anyway because of the pacemaker, and I don’t want to cause no, don’t have no heart attack”. (Veteran 26)*

## Summary

- Key points
  - Approximately 250,000 adults aged 65 years and older undergo inguinal hernia repair annually in the United States, but there is a considerable lack of understanding of how hernia surgery affects the day-to-day lives of older adults.
  - Older adults desire improved listening and clearer communication regarding the reality of surgical recovery.
  - Robust preoperative counseling that focuses on the reality of postoperative recovery and life after hernia surgery may improve patient satisfaction.
- Why does this paper matter?
  - This paper provides specific and actionable ways that surgeons can improve the quality of preoperative communication and enhance postoperative recovery from hernia surgery.
